# Advances in Urological Cancer in 2022, from Basic Approaches to Clinical Management

**DOI:** 10.3390/cancers15051422

**Published:** 2023-02-23

**Authors:** Claudia Manini, Estíbaliz López-Fernández, José I. López, Javier C. Angulo

**Affiliations:** 1 Department of Pathology, San Giovanni Bosco Hospital, 10154 Turin, Italy; 2 Department of Sciences of Public Health and Pediatrics, University of Turin, 10124 Turin, Italy; 3 FISABIO Foundation, 46020 Valencia, Spain; 4 Faculty of Health Sciences, European University of Valencia, 46023 Valencia, Spain; 5 Biocruces-Bizkaia Health Research Institute, 48903 Barakaldo, Spain; 6 Clinical Department, Faculty of Medical Sciences, European University of Madrid, 28005 Madrid, Spain; 7 Department of Urology, University Hospital of Getafe, 28907 Madrid, Spain

This Special Issue includes 12 articles and 3 reviews dealing with several basic and clinical aspects of prostate, renal, and urinary tract cancer published during 2022 in *Cancers*, and intends to serve as a multidisciplinary chance to share the last advances in urological neoplasms. 

This international forum of urological cancer includes different perspectives from 14 different countries: Canada, New Zealand, Italy, the USA, Germany, South Korea, Japan, Spain, Austria, China, the Netherlands, Taiwan, Poland, and the UK. An overview of these contributions shows the great variability of topics currently impacting the urological clinical practice, from the molecular mechanisms underlying prostate cancer development, for example, to the appropriateness of the robotic surgery in radical prostatectomy or partial nephrectomy and the current role of prostate-specific membrane antigen positron-emission tomography (PSMA-PET) imaging in prostate cancer. In addition, this “Urological Cancer 2022” Issue is also an opportunity to highlight some relevant international achievements of the specialty published elsewhere during 2022.

Pellerin et al. [1] analyzed the effects of the chronic exposition to bisphenols in bladder epithelium. Bisphenols A and S are chemical compounds used in the plastic industry to produce polycarbonates necessary for generating epoxy and vinyl ester resins. These worldwide distributed composites are industrially produced by the condensation of phenol and acetone and make up part of several plastics such as PVC. These products are insoluble in water and are present in the urine in normal conditions. Importantly, they are endocrine disruptors that interfere with cellular signaling pathways in urothelial cells [2]. Using normal urothelial cells (pediatric volunteers) and non-invasive (RT4, cell line ATCC HTB-2) and invasive (T24, cell line ATCC HTB-4) bladder cancer cells, the authors evaluate the impact of bisphenols on the energy metabolism, proliferation, migration, and pro-tumorigenic effect in human urothelium. They conclude that a chronic exposure to bisphenols A and S increases the proliferation rate and decreases the migration capacities of normal urothelial cells, which could result in urothelial hyperplasia. By contrast, these chemical products increase the energy metabolism, physiological activity, and cell proliferation, which could eventually promote urothelial cancer progression, especially from non-invasive to invasive variants.

The clinical identification of aggressive variants of prostate carcinoma requires more accurate markers. Reader et al. [3] have analyzed how the variations in the expression of Activins B and C impact the growth of PNT1A and PC3 prostate cancer cell lines. Activins are homo- or hetero-dimers belonging to the transforming growth factor-β family involved in prostate homeostasis, which are dysregulated in prostate cancer [4]. The authors have detected that the expression of Activin B was increased in prostate cancer samples, with a higher Gleason index, and that its overexpression inhibited the growth of PNT1A cells and increased PC3 cells’ growth and migration. Interestingly, Activin C showed the opposite expression, with decreased immunostainings in prostate cancer cells with high Gleason grades, an increased overexpression in PNT1A cells, and a decreased growth in PC3 cells. The authors conclude that the combination of Activin B increasing and Activin C decreasing is associated with a higher Gleason grade in prostate adenocarcinoma and suggest its potential usefulness as prognostic biomarkers in this neoplasm.

Tossetta et al. [5] focused on the role of the ciliary neurotrophic factor (CNTF) in prostate cancer. Despite the fact that several crucial pathways such as MAPK/ERK, AKT/PI3K, and ^Jak^/STAT regulate prostate cancer progression are triggered by CNTF, little is known about the effect of this member of the IL-6 family. The authors analyze the immunohistochemical expression of CNTF and its receptor in androgen-responsive (*n* = 10, radical prostatectomy samples) and castration-resistant (*n* = 10, transurethral resection samples) prostate cancers. Additionally, CNTF and its receptor expression are analyzed in androgen-dependent (LNCaP) and androgen-independent (11Rv1) prostate cancer cell lines by Western blotting and immunofluorescence. They also show that CNTF treatment downregulates MAPK/ERK and AKT/PI3K pathways, inhibiting the matrix metalloproteinase-2 (MMP-2), a major component of the extracellular matrix degrader, and what is mainly responsible for tumor invasiveness. The authors conclude that CNTF plays a key role in the remodeling of the prostate cancer environment and suggests that this cytokine may modulate prostate cancer invasion. CNTF could represent a novel therapeutic approach in patients with castration-resistant prostate cancer.

Zapala et al. [6] investigated the usefulness of the preoperative systemic immune-inflammation index (SII) in predicting survival in a retrospective series of 421 patients with non-metastatic prostate cancer treated with radical prostatectomy. They found that a high SII was an independent predictor of overall survival. Furthermore, the combination of high age-adjusted Charlson Comorbidity Index (ACCI), the Cancer of the Prostate Risk Assessment Postsurgical score (CAPRA-S), and the SII identifies patients at the highest risk of death. The authors conclude that SII should be added to the prognosticators of patients with prostate cancer.

Clear cell renal cell carcinoma (CCRCC) is a perfect example of tumor complexity and a permanent target of analysis in recent years. In this collection, Shi et al. [7] analyzed the value of a ferroptotic gene-based signature in the prognosis of several series of CCRCC downloaded from the GEO database. The analyzed genes were obtained from the FerrDb V2 database. They identify a set of nine genes with prognostic implications differentially expressed in CCRCC, and found that the GLS2 enzyme, encoded by the *GLS2* gene and regulated by p53, may be a ferroptotic suppressor in CCRCC. The authors conclude that this nine-gene signature could eventually be an independent prognosticator in this neoplasm and advice for further investigations. Aside from that, several interesting investigations have been performed this year and deserve a short mention. Intratumor heterogeneity (ITH) is a constant, extensively analyzed event in CCRCC and its level has been correlated in 2022 with tumor aggressiveness. A mathematical study based on game theory [8] and a histological analysis [9] confirm that aggressive variants of CCRCC typically display low levels of ITH and agree with a genomic analysis of 101 cases already published in 2018 [10]. Additionally, several investigations have analyzed the influence of tumor growth patterns in the inter-regional genomic variability of these neoplasms [11], supporting the need for a personalized tumor sampling to strengthen tumor analysis [12,13].

In their review, Christenson et al. [14] revisited all the treatment modalities available so far in prostate cancer, focusing especially on the targets to interrupt the biological progression in lethal forms of prostate cancer, that now have a 5-year overall survival of only 30%. They revise current therapies, considering first low-risk and high-risk non-metastatic cancer, and then how to target metastatic cases, including hormone therapy, chemotherapy, PSMA-targeted radiation, genome-targeted precision therapy, and immunotherapy. The authors also review the ETS fusion positive (involving *TMPRSS2*, *SLC45A3*, *ETV1*, *ETV4*, and *FLI1* genes) and negative (involving *SPOP*, *FOXA1*, and *IDH1* genes) molecular subtypes of prostate cancer. The intimate mechanisms regulating metastatic castration-resistant prostate cancer and the androgen receptor ablation for intercepting advanced cancer are also analyzed. The intestinal microbiota as a potential promoter of castration-resistant tumor variants, the innovations in managing neuroendocrine tumors, and the difficulties of applying immune checkpoint inhibition in prostate cancer appear among the future directions in the review. Finally, the authors point also to CRISPR/Cas enzymes-assisted gene editing as a promising arena to develop further in prostate cancer management.

Other clinically oriented topics have also been incorporated in this Special Issue on the advances in urologic cancer regarding patient follow-up, diagnosis, and treatment. A very interesting one is a randomized clinical trial performed in the Netherlands which deals with the transition of care between a specialist and primary care physician [15]. Patients were randomized and allocated to specialist or general practitioner care for a head-to-head comparison. Several advantages of primary care follow-up over specialists’ have been identified, including accessibility and more personalized attention, with a similar effectiveness. This study also identifies several challenges that must be addressed before the transition to primary-care follow-up can be a reality, by using quality indicators and improving communication and collaboration. However, another report from the same study shows that from the patient’s perspective, hospital-based follow-up is preferred, but efforts should be made to improve physician’s knowledge about personal aspects of the patient, improve symptoms management, and promote global health [16].

A series of articles evaluate the diagnostic pitfalls of prostate cancer by using different tools including PSA, multiparametric magnetic resonance imaging (mpMRI), and new generation imaging with the PSMA-PET modality; the latter has many therapeutic implications. In this respect, a study addresses the variables associated with false-positive PSA results using real-world data in a Spanish cohort of 1664 patients followed for two years. The false-positive results were as high as 47%, resulting in a positive predictive value of merely 13% [17]. This rate is much higher than previously reported in trials with screening data [18] Many factors were demonstrated to be associated with the presence of false-positive results, including age, previous PSA evaluation, family history of prostate cancer, and alcohol intake, but these associations were sustained only in asymptomatic patients [17]. These data do not serve to evaluate overdiagnoses and overtreatment but help to sustain that the PSA era in prostate cancer diagnosis should be closing.

A study in this Special Issue addresses the limitations of mpMRI for primary prostate cancer diagnosis in the form of a systematic review and meta-analysis that compare mpMRI with prostate-specific membrane antigen (PSMA) positron emission tomography (PET) imaging [19]. With the limitation of significant heterogeneity observed, PSMA-PET computerized tomography (CT) seems superior to mpMRI in primary cancer diagnosis, but not in the definition of cancer location within the gland. However, as can be expected from a whole-body procedure, PSMA-PET CT has valuable potential for tumor staging. False-positive MRI is another troublesome reality in clinical practice as it is not easy to differentiate false and real positive lesions, thus making evident the difficulty of further advancing in the MRI diagnostic ability [20]. PSMA-PET CT has also been investigated to solve MRI Prostate Imaging Reporting and Data System (PI-RADS) 4–5 lesions and negative biopsy discordance [21] and also the combination of PSMA-PET CT and mpMRI is being currently investigated in the MP4 clinical trial in which PI-RADS 4 or 5 lesions ≥10 mm on mpMRI are given the option of a PSMA-PET CT before biopsy. The intention of this approach is to predict better aggressive prostate cancer. That opens a new perspective to evaluate the feasibility of proceeding to prostate cancer surgery directly without a biopsy [22]. Additionally, PROSPET-BX clinical trial, currently undertaken in Italy, might confirm the superiority of PSAM-PET CT/transrectal ultrasound (TRUS) fusion prostate biopsy over mpMRI/TRUS fusion biopsy to further spare unnecessary biopsies [23].

Inspired by all these changes of paradigm, an excellent review on the clinical applications of PSMA-PET CT examination in patients with prostate cancer has been included in this Special Issue [24]. The article addresses the limitations and pitfalls of this new generation diagnostic imaging modality and emphasizes its therapeutic implications also. In fact, prolonged progression free and overall survival has been very recently confirmed in castration-resistant prostate cancer with lutetium Lu 177 vipivotide tetraxetan (^177^Lu-PSMA-617) radioligand therapy [25]. Additionally, the PSMA-PET CT-guided intensification of radiotherapy is being investigated in a Canadian clinical trial, with special effort on cancer control, long-term toxicity, and health-related quality of life issues [26].

Carbon-ion radiotherapy, another modality to improve the effectiveness of radiation therapy, has been also evaluated in this Special Issue, with a retrospective study focusing on the older population of patients with prostate cancer [27]. This study confirms that carbon-ion radiotherapy is a safe and effective high-dose intensive treatment. This modality of radiation has been popular in different institutions in Japan for the treatment of different urologic cancers [28]. This modern technology provides several unique physical and radiobiologic properties that allow low levels of energy to be deposited in tissues proximal to the target, while the majority of energy is released in the target itself. That may have important advantages, especially in the setting of recurrent disease [29]. Another modality of radiation is extreme hypofractionation with stereotactic body radiation therapy (SBRT) in which treatment is delivered in one to five fractions, an encouraging alternative in the low- and intermediate-risk profile of patients that competes with high-dose brachytherapy [30,31].

Surgery has also seriously evolved to consider robotic prostatectomy the gold standard of surgical care for localized prostate cancer, that improves the functional outcomes of urinary continence and potency. This is also the topic of another article in the Special Issue [32]. Still, the definition of continence “without pads” or “social continence” makes difficult the comparison of the results [33]. New and effective modalities to surgically correct post-prostatectomy incontinence have been developed in recent decades and can be used both for stress urinary incontinence after prostatectomy and after radiation therapy [34,35,36].

Another interesting application of robotics in surgical urologic oncology is partial nephrectomy. The clinical benefits of indocyanine green florescence in robot-assisted partial nephrectomy are discussed in another element of this Special Issue. Reduced blood loss without a negative impact in the positive surgical margin rate is suggested using green dye [37]. However, future prospective randomized controlled trials are needed to confirm the presumed operative and functional advantages of this approach. Moreover, the issue presents another very interesting collaboration regarding metastatic renal cell carcinoma treatment, a field that has been subject to important paradigm changes in recent years [38]. The German multicenter prospective study PAZOREAL presented by Doehn et al. [39] reveals very interesting data on the effectiveness and safety of pazopanib (first-line), nivolumab (second-line), and everolimus (second- and third-line) in a real-life setting. This sequence is widely used in clinical practice. Targeted treatments for metastatic renal cell carcinoma allow for a more tailored approach, but predictive elements for immune-checkpoint inhibitors or tyrosine kinase inhibitors as a first-line treatment still lack genuine prediction markers [40].

Many studies have faced the optimal management of bladder urothelial malignancy in recent years. Some have searched for new therapeutic alternatives in the scenario of Bacillus Calmette-Guerin (BCG) shortage to prevent urothelial cancer recurrence and progression. Device-assisted intravesical chemotherapy using recirculating hyperthermic mitomycin-C (HIVEC) has been widely used in Spain [41]. Current new evidence favors the use of HIVEC in high-risk non-muscle-invasive bladder cancer [42], but not in the intermediate risk [43]. Many other studies have extended to step beyond classical morphologic parameters and stratify the prognosis of muscle-invasive bladder cancer according to new molecular markers that take into account basal or luminal phenotypes discovered [44,45]. A further step that is currently being undertaken is the evaluation of the intratumor microenvironment landscape, with implications not only in prognosis, but also in the response to systemic immunotherapy [46]. In this sense, immune-checkpoint inhibitors have been recently approved as a second-line treatment for metastatic bladder cancer and are currently being investigated in a neoadjuvant setting in non-metastatic disease [47,48].

Upper urinary tract urothelial carcinoma is another malignancy addressed in the Special Issue. Ha et al. revealed that intravesical recurrence after radical nephroureterectomy is associated with flexible but not with rigid diagnostic ureteroscopy [49]. This specific report opens a new perspective that requires a further evaluation in large-population controlled studies. The issue of intravesical recurrence after upper urinary tract cancer diagnosis and treatment is a big unsolved problem in the comprehensive management of urothelial malignancy. Several meta-analyses have confirmed the higher rate of intravesical recurrence after radical nephroureterectomy in patients who underwent diagnostic ureteroscopy preoperatively [50,51,52], but with no concurrent impact on long-term survival [52]. Probably the negative impact on intravesical recurrence free survival is due more to endoscopic biopsy than to ureteroscopy itself. As the use of flexible ureteroscopy is routinely recommended by clinical guidelines [53], future studies are needed to assess the role of immediate postoperative intravesical chemotherapy in patients undergoing biopsy during ureteroscopy for suspected upper tract urothelial cancer.

In summary, “Urological Cancer 2022” is a remarkable piece of knowledge that presents new relevant clinical, molecular, imaging, and therapeutic data in the urological field and invites researchers in urologic malignancy to enter a multidisciplinary approach and face some of the most relevant and current topics in urology.
